# Effects of spironolactone and losartan on the early neovascularization of acute myocardial infarction

**DOI:** 10.3892/etm.2014.1791

**Published:** 2014-06-18

**Authors:** YAN LIU, KUNSHEN LIU

**Affiliations:** 1 Department of Geriatrics, The First Hospital of Shijiazhuang City, Shijiazhuang, Hebei 050011, P.R. China; 2 Department of Cardiology, The First Affiliated Hospital of Hebei Medical University, Shijiazhuang, Hebei 050031, P.R. China

**Keywords:** acute myocardial infarction, spironolactone, losartan, capillary density, matrix metalloproteinase

## Abstract

The aim of the present study was to investigate the effects of spironolactone and losartan on the early healing stage of acute myocardial infarction (AMI) in rats. An AMI rat model was established and the rats were randomly divided into four groups: AMI (n=12), AMI + spironolactone (AMI + S; n=12), AMI + losartan (AMI + L; n=12) and AMI + spironolactone combined with losartan (AMI + S + L; n=12). Sham-operated rats served as a control group (n=12). The expression levels of matrix metalloproteinases (MMPs) and tissue inhibitors of matrix metalloproteinases (TIMPs) in the non-infarcted myocardium surrounding the AMI area were determined using immunohistochemistry. In addition, the capillary density in the non-infarcted myocardium surrounding the AMI area was detected. The capillary densities around the infarcted area in the AMI and treatment groups at day 7 and 14 following AMI surgery were significantly higher compared with the sham-operated rats. Compared with the AMI group, the capillary densities around the infarcted area and the ratio of MMPs/TIMP-1 were increased in the treatment groups following AMI surgery; however, the increased ratio of MMPs/TIMP-1 was reduced at day 14 following AMI surgery. Therefore, these results indicated that spironolactone and losartan may promote the formation of collateral circulation in the non-infarcted tissue surrounding the infarcted area by regulating the production of MMPs.

## Introduction

Numerous studies have demonstrated that aldosterone is an important etiology of myocardial fibrosis and cardiac remodeling following myocardial infarction (1–4). Clinical trials conducted by the Randomized Aldactone Evaluation Study (5) and Eplerenone Post-Acute Myocardial Infarction Heart Failure Efficacy and Survival Study (6) have demonstrated that the receptor antagonists of aldosterone may significantly reduce cardiovascular-associated mortality in patients with severe heart failure. Recent studies have also shown that the receptor antagonists of mineralocorticoids may improve the survival of patients with systolic heart failure or mild to severe symptoms of heart failure (7–9). Clinical data have indicated that angiotensin-converting enzyme inhibitors are an effective therapeutic strategy for the treatment of heart failure to inhibit aldosterone and improve cardiac remodeling. However, due to their side effects, including an increase in the incidence of cough of ≤26%, these drugs are not suitable for certain patients. Angiotensin receptor blockers selectively inhibit angiotensin 1 (AT1) and completely inhibit angiotensin II (Ang II). They exhibit similar efficacy and cardiac- and vascular-protective effects as angiotensin-converting enzyme inhibitors (10,11). Therefore, these drugs may be used as an alternative for angiotensin-converting enzyme inhibitors in the treatment of heart failure.

Left ventricular remodeling is an important aim in the treatment of acute myocardial infarction (AMI) and heart failure (12). The degree of left ventricular remodeling following AMI is closely associated with severe left ventricular dysfunction and progression to congestive heart failure. The structure and function of the extracellular matrix (ECM) is maintained by matrix metalloproteinases (MMPs); however, degradation of the ECM is regulated by the endogenous tissue inhibitors of matrix metalloproteinases (TIMPs). Excessive degradation of components of the ECM leads to the expansion of the left ventricle and heart failure. Since TIMP-1 has a high affinity to a number of MMPs, binding to MMPs can inhibit the proteolytic activity of MMPs and have a direct role in left ventricular remodeling, indicating that TIMPs may be used to predict the clinical outcomes of patients with heart failure (13,14). TIMPs form MMP-TIMP complexes with MMPs in a 1:1 ratio to inhibit the binding of MMPs to their substrates. If the ratio of MMPs/TIMP-1 increases, the degradation of the ECM is accelerated. MMP-2 and MMP-9 are two gelatinases that degrade type IV and type V collagens. However, the degradation of type IV and V collagens is an indication of inflammation-mediated angiogenesis.

Michel *et al* (15) demonstrated that aldosterone may be used to induce the formation of new blood vessels in ischemic tissues via the Ang II pathway, which was not completely inhibited by 20 mg/day/kg spironolactone, a receptor antagonist of aldosterone, or 20 mg/day/kg valsartan, a type I receptor antagonist of Ang II. In the present study, the effect of higher doses of aldosterone receptor antagonist and AT1 receptor antagonist on the angiogenesis in the surrounding tissues of an AMI area was investigated. An AMI rat model was treated with a large dose of spironolactone (80 mg/day/kg) and/or losartan (10 mg/day/kg) to investigate the changes in the expression levels of MMP-2, MMP-9 and TIMP-1, as well as the capillary density, in the non-infarcted area surrounding the infarcted area. The aim of the study was to observe the effects of spironolactone and/or losartan on the early neovascularization in AMI rats.

## Materials and methods

### Animals

A total of 60 healthy male Wister rats, weighing between 220 and 250 g, were obtained from the Experimental Animal Center of Hebei Medical University (Shijiazhuang, China). The rats were randomly divided into sham-operated (n=12) and AMI groups (n=48). The animals used in the study were handled and treated in accordance with the strict guiding principles of the National Institutes of Health for the Experimental Care and Use of Animals. The experimental design and procedures were approved by the Institutional Ethical Committee for Animal Care and Use of Hebei Medical University.

### Establishment of the AMI rat model and treatment

In the AMI group, the left anterior descending coronary artery of the rats was ligated to induce a large area of AMI. Sham-operated rats underwent the same surgery, but without ligation. Following surgery, all the animals were housed alone at a constant temperature of 37°C. After 72 h, the surviving rats in the AMI group were randomly divided into four groups and treated as follows. The AMI group (n=12) were treated with physiological saline. The AMI + spironolactone group (AMI + S group, n=12) were administered 80 mg/day/kg spironolactone intragastrically, while the AMI + spironolactone + losartan group (AMI + S + L group, n=12) were administered 80 mg/day/kg spironolactone and 10 mg/day/kg losartan intragastrically. The AMI + losartan group (AMI + L group, n=12) were administered 10 mg/day/kg losartan intragastrically and the sham-operated group were administered an equal volume of physiological saline.

### Sampling

At day 7 and 14 following AMI surgery, six rats in each group were anesthetized with 10% chloral hydrate, and the heart was quickly removed. The hearts were rapidly placed into 4% paraformaldehyde solution and fixed for 12–24 h. Following dehydration using gradient concentration (70%, 80%, 95% and 100%) of alcohol-water solution, the heart tissues were embedded in paraffin blocks. Serial sections of 5 μm-thickness were mounted on poly-L-lysine-coated slides and used for hematoxylin and eosin (HE) and immunohistochemical staining of TIMP-1, MMP-2 and MMP-9.

### Immunohistochemical analysis [Streptavidin peroxidase (SP) method]

Heart tissue sections were deparaffinized to hydrophilia and incubated with 3% H_2_O_2_ in methanol for 10 min at room temperature to remove the endogenous peroxidase activity. The sections were rinsed with distilled water and 0.01 M phosphate-buffered saline (PBS; pH 7.4). Slides were microwaved in 0.1 M citrate buffer (pH 6.0) at 98°C for 20 min and then cooled to room temperature, which was followed by incubation with 10% normal rabbit serum in 0.1 M PBS (pH 7.4) to block the non-specific sites at room temperature for 30 min. Respective incubation with rabbit anti-rat TIMP-1, goat anti-rat MMP2 and MMP9 (all diluted 1:50 in PBS; Beijing ZSGB-Biotechnology, Co., Ltd., Beijing, China) was performed overnight at 4°C in a humidified chamber, prior to incubation with secondary biotin-conjugated antibodies and sequential incubation with horseradish peroxidase-conjugated streptavidin and the peroxidase substrate, 3′-diaminobenzidine (all from Beijing ZSGB-Biotechnology, Co., Ltd., Beijing, China). Samples were then counterstained with hematoxylin. PBS was used instead of a primary antibody as a negative control.

### Evaluation of immunohistochemical staining

Positive immunoreactions for TIMP-1, MMP-2 and MMP-9 presented with brown or yellow staining in the cytoplasm. Under a microscope (Microphot-FXA, Nikon Corp., Shizuoka, Japan), 10 randomly selected fields in each slide were observed and 15 positive sites in each field were imaged in order to calculate the gray values using imaging analysis system software (Beihang University, Beijing, China). The mean gray values of each slide were calculated and compared.

### Determination of capillary density

Slides with HE staining were observed under a light microscope. The number of capillaries in 10 randomly selected fields (magnification, ×200) in each section were calculated. Results were expressed as the average of the numbers/mm^2^.

### Statistical analysis

Results are expressed as the mean ± standard deviation. Normal distribution of the data was first verified. If normal, comparisons between the groups were performed using one-way analysis of variance. Comparisons between the sham-operated and AMI groups were conducted using the least significant difference test, while comparisons between any two groups were performed using the Student-Newman-Keuls test. All statistical analysis was performed using SPSS 10.0 software (SPSS, Inc., Chicago, IL, USA), and P<0.05 was considered to indicate a statistically significant difference.

## Results

### Effect of spironolactone and/or losartan on angiogenesis following AMI

At day 7 and 14 following AMI surgery, the rats treated with spironolactone and/or losartan presented higher capillary density in the non-infarcted tissue around the infarcted area when compared with the AMI or sham-operated groups (Table I).

### MMP-2 expression in the non-infarcted tissue around the infarcted area

At day 7 following AMI surgery, only the AMI + S group exhibited higher expression levels of MMP-2 in the non-infarcted tissue surrounding the infarcted area when compared with the AMI group (P<0.01). However, at day 14 following AMI surgery, the increased expression of MMP-2 was reduced significantly compared with the AMI group (P<0.01; Table I).

### MMP-9 expression in the non-infarcted tissue around the infarcted area

At day 14 following AMI surgery, the three treatment groups exhibited significantly lower expression levels of MMP-9 in the non-infarcted tissue around the infarcted area when compared with the AMI group (P<0.01), and similar levels to that of the sham-operated group (Table I).

### Changes in the expression levels of TIMP-1 in the non-infarcted tissue around the infarcted area following treatment

TIMP-1 expression levels in the non-infarcted tissue around the infarcted area were markedly lower in the three treatment groups at day 7 following AMI surgery when compared with the AMI and sham-operated groups (P<0.01). However, at day 14 following AMI surgery, the expression increased gradually and the levels were higher than those in the AMI group (P<0.01) and close to the level in the sham-operated group (Table I).

### MMPs/TIMP-1 ratio in the non-infarcted tissue around the infarcted area was significantly affected by spironolactone and/or losartan

Compared with the AMI group, the ratios of MMPs/TIMP-1 in the three treatment groups were increased at day 7 following AMI surgery; however, the ratio then increased to a normal level at day 14 following AMI surgery (Table II).

## Discussion

Aldosterone has been previously demonstrated to significantly enhance ischemia-induced angiogenesis via the Ang II pathway (1) and increase the expression of vascular endothelial growth factor by 2.3-fold. However, 20 mg/day/kg spironolactone or 20 mg/day/kg valsartan does not completely inhibit this effect. Thus, the present study investigated the effects of higher doses of aldosterone receptor antagonist and AT1 receptor antagonist in the inhibition of vascularization in the surrounding area of the infarcted area. The effects of high doses of the aldosterone receptor antagonist, spironolactone (80 mg/day/kg), and the AT1 receptor antagonist, losartan (10 mg/day/kg), were analyzed in the early stages of vascularization in AMI rats. The results demonstrated that high doses of spironolactone (80 mg/day/kg) and losartan (10 mg/day/kg) significantly increased the ratios of MMPs/TIMP-1 in the non-infarcted tissue surrounding the infarcted area in AMI rats at day 7 following AMI surgery. However, the ratios were reduced to a normal level at day 14 following AMI surgery. The application of spironolactone and losartan may also enhance the capillary density in the non-infarcted tissue surrounding the infarcted area in AMI rats, indicating that spironolactone and losartan have a promotive effect on angiogenesis in the non-infarcted tissue surrounding the infarcted area in AMI rats.

Selective degradation of the ECM to adjust the morphology, growth and survival of vascular endothelial cells can affect the growth and degradation of capillaries during angiogenesis (16), while the selective secretion and activation of MMPs indicates the changes in cell-cell junctions and the extension of cells. MMP-2 and MMP-9 are two gelatinases that hydrolyze collagen IV and collagen V. If collagen IV and V are degraded, the ECM molecules change, activating vascular endothelial cells via intracellular signaling pathways, which promotes the formation of neovessels. Johnson *et al* (17) demonstrated that the capillary density in the ischemic tissue of MMP-9−/− mice was not significantly different compared with the density prior to AMI surgery, while the capillary density in the ischemic tissue of wild-type mice increased by 39%. These observations indicate that activation of MMP-9 is essential for a variety of physiological and pathological processes in the angiogenesis of ischemic tissues. Yan *et al* (18) found that reducing the cleavage of pro-MMP-2 increased fibronectin density in the cultured endothelial cells and caused cells to adhere and extend, indicating that MMP-2 may regulate vascular endothelial cells. A study by Haas *et al* (19) demonstrated that the hydrolysis of the basement membrane proteins of capillaries is one indication of inflammatory-mediated angiogenesis. TIMPs are specific endogenous inhibitors of MMPs. Dynamic alteration in the ratio of MMPs/TIMPs may result in the selective degradation of ECM, thereby affecting the growth and degradation of capillaries during angiogenesis.

The effects of spironolactone and/or losartan on neovascularization in the non-infarcted tissue surrounding the infarcted area were also investigated in the present study. The expression levels of MMP-2, MMP-9 and TIMP-1 were determined in the non-infarcted tissue surrounding the infarcted area of the AMI rats treated with or without spironolactone and/or losartan. In addition, the ratios of MMPs/TIMP-1 in the non-infarcted tissue surrounding the infarcted area of AMI rats were increased by spironolactone and/or losartan at day 7 following AMI surgery; however, the ratios then reduced to normal levels at day 14 following AMI surgery. These results indicate that the application of spironolactone and losartan in the early stages of AMI may promote angiogenesis in the non-infarcted tissue surrounding the infarcted area of AMI rats via dynamic changes between the levels of TIMP-1 and MMP-2 and MMP-9. The elevated ratios of MMP-2/TIMP-1 and MMP-9/TIMP-1 accelerated the degradation of non-fibrous collagen in the basement membrane, thereby inducing angiogenesis and promoting the formation of collateral circulation.

The production of aldosterone and Ang II in the adrenal glands and non-infarcted myocardium of AMI rats has been shown to be significantly increased. In addition, aldosterone has been demonstrated to significantly enhance the induction of ischemia on the formation of new blood vessels via the Ang II pathway. However, high doses of spironolactone (80 mg/day/kg) did not inhibit the proinflammatory effects of aldosterone (20). Silvestre *et al* (20) found that the mRNA expression of aldosterone synthase and the levels of Ang II were significantly higher in the non-infarcted area of AMI rats that had been treated with 80 mg/day/kg spironolactone at day 25 following AMI surgery. These observations may be the result of negative feedback or that aldosterone did not entirely exert its effects, including proinflammatory, promotion of tissue-repairing and enhancing ischemia-induced angiogenesis, via the aldosterone receptors or AT1 receptors. However, the exact mechanism remains to be elucidated.

Drüppel *et al* (21) demonstrated that aldosterone induced stiffed endothelial cell syndrome (SECS), characterized by the upregulation of epithelial sodium channels (ENaCs) and the mechanical enhancements of the endothelial cell cortex, combined with vascular endothelial dysfunction. *In vivo*, the antagonism of aldosterone has a continual protective role on the cardiovascular system. However, in cultured endothelial cells, the expression of the mineralocorticoid receptor was increased by 129%. The expression of ENaCs increased by 32 and their surface area increased by 42% (from 13.8 to 19.6), respectively. However, the receptor was inhibited by spironolactone. Following three weeks of treatment, the release of nitric oxide was elevated by 50%. Thus, spironolactone was shown to prevent the time-dependent performance of SECS by long-lasting improvement of endothelial function, which emphasizes the critical role of vascular endothelial cells as a therapeutic target in cardiovascular disease.

In conclusion, the results of the present study demonstrated that the application of high doses of aldosterone receptor antagonist and AT1 receptor antagonist during the early stages following AMI surgery is able to inhibit angiogenesis in the surrounding area. However, the optimal dose and time for the early application of spironolactone and the exact effects on cardiac function require further investigation, as well as the mechanisms underlying the function of aldosterone receptor antagonists in AMI.

## Figures and Tables

**Table I:** Expression levels of MMP-2, MMP-9 and TIMP-1, and the capillary density of each group.

Groups	MMP-2	MMP-9	TIMP-1	Capillary density, capillaries/mm^2^
Sham-operated				
Day 7	0.166±0.015	0.172±0.016	0.231±0.014	13.384±0.293
Day 14	0.153±0.015	0.171±0.016	0.236±0.014	13.426±0.226
AMI				
Day 7	0.184±0.013	0.203±0.014	0.228±0.014	14.574±0.374
Day 14	0.162±0.013	0.191±0.015	0.199±0.018	14.482±0.262
AMI + S				
Day 7	0.197±0.013	0.192±0.015	0.207±0.016	15.754±0.464
Day 14	0.146±0.012	0.178±0.013	0.236±0.016	20.225±0.513
AMI + L				
Day 7	0.176±0.012	0.186±0.016	0.204±0.018	18.575±0.266
Day 14	0.151±0.093	0.172±0.085	0.225±0.014	19.483±0.403
AMI + S + L				
Day 7	0.182±0.088	0.206±0.016	0.197±0.014	16.834±0.437
Day 14	0.153±0.087	0.174±0.013	0.236±0.015	20.455±0.405
F-value	58.72	60.58	34.51	110.84
P-value	<0.01	<0.01	<0.01	<0.01

Data are expressed as the mean ± standard deviation. MMP, matrix metalloproteinase; TIMP, tissue inhibitor of matrix metalloproteinase; AMI, acute myocardial infarction; S, spironolactone; L, losartan. The F and P-values refer to the comparison result of day 14 following AMI surgery among multiple groups using one-way analysis of variance.

**Table II:** Ratios of MMP-2/TIMP-1 and MMP-9/TIMP-1 in each group.

Parameters	Sham-operated	AMI	AMI + S	AMI + L	AMI + S + L
MMP-2/TIMP-1					
Day 7	0.719	0.807	0.952	0.863	0.924
Day 14	0.648	0.814	0.619	0.671	0.648
MMP-2/TIMP-1					
Day 7	0.745	1.009	0.952	0.912	1.046
Day 14	0.725	0.962	0.754	0.764	0.737

MMP, matrix metalloproteinase; TIMP, tissue inhibitor of matrix metalloproteinase; AMI, acute myocardial infarction; S, spironolactone; L, losartan.
